# Continuous intravenous to oral morphine switch in very premature ventilated infants: A retrospective study on efficacy, efficiency, and tolerability

**DOI:** 10.1002/pne2.12011

**Published:** 2020-01-03

**Authors:** Phoï Duong, Manon Tauzin, Fabrice Decobert, Laetitia Marchand, Laurence Caeymaex, Xavier Durrmeyer

**Affiliations:** ^1^ Neonatal Intensive Care Unit CHI Créteil Créteil France; ^2^ CRESS INSERM 1153 INRA Université de Paris Paris France; ^3^ Faculté de Médecine de Créteil Université Paris Est Créteil IMRB, GRC CARMAS Créteil France

**Keywords:** intravenous morphine, mechanical ventilation, oral morphine, pain, very premature infants

## Abstract

**Background:**

Continuous intravenous (IV) morphine is commonly used in ventilated neonates. Oral route is theoretically feasible but data on oral morphine in ventilated premature infants are lacking.

**Objective:**

To assess the efficacy, efficiency, and tolerability of a continuous intravenous to oral morphine switch protocol.

**Design:**

Retrospective study.

**Setting:**

Single level III center's neonatal intensive care unit.

**Patients:**

Ventilated premature infants hospitalized in the NICU in 2016 and 2017, receiving continuous IV morphine with an expected ventilation course of at least 72 more hours. We excluded patients treated for withdrawal syndrome or palliative care.

**Interventions:**

Continuous IV to oral morphine switch with the same initial cumulated daily dose.

**Main outcome measures:**

Pain scores (ComfortNeo scale) and morphine doses were analyzed over time using Friedman's test in the 24 hours preceding and the 48 hours following the oral switch. Adverse effects attributable to opioids were collected.

**Results:**

Seventeen infants were included with a median [IQR] gestational age at birth of 25.9 [24.6‐26.9] weeks and a median postnatal age at oral switch of 30 [22‐36] days. One patient's intravenous treatment had to be resumed because of a high ComfortNeo score. All others remained on oral morphine. No significant change over time was observed for ComfortNeo scores (*P* = .15). Median [IQR] doses were 13.5 [10‐20] µg/kg/h in the IV period and significantly increased to 15 [10‐25] µg/kg/h in the oral period (*P* = .009). No short‐term respiratory, digestive, or urinary adverse event was observed. After a median [IQR] duration of 13 [4‐20] days of oral morphine treatment, 11 (65%) patients showed signs of withdrawal. Upon hospital discharge, 16 infants (94%) had bronchopulmonary dysplasia and none had severe cerebral abnormality on brain imaging.

**Conclusion:**

Oral morphine might be useful in ventilated neonates in the NICU but deserves further studies and additional safety assessment.

## Graphical abstract

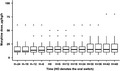

## 1 INTRODUCTION

Neonatal clinical pharmacology is particularly challenging because of the great inter‐individual variability in pharmacodynamics and pharmacokinetics as well as intra‐individual variability depending on gestational age at birth, postnatal age, diseases, and associated treatments.^1^ Adequate pain monitoring and control in premature infants are very important, as exposure to repeated painful stimuli early in life has been associated with adverse neurodevelopmental, behavioral, and cognitive outcomes.^2^

To control neonatal pain in the neonatal intensive care unit (NICU), morphine is the most commonly used opioid.^3^ Currently, it is recognized that doses of continuous intravenous (IV) morphine between 5 and 20 µg/kg/h effectively reduce signs of pain in intubated premature or term neonates.^4^, ^5^ Follow‐up studies of ventilated neonates included in a double‐blind, randomized placebo‐controlled trial of morphine (10 µg/kg/h) found conflicting results over time with negative effect of morphine on cognitive function at age 5 6 and positive effect on the executive functioning at school age.^7^ On the other hand, associations have been reported between cumulative morphine doses in the neonatal period and poor neurodevelopmental outcome at 18 or 24 months.^8^, ^9^ Thus, efforts to achieve the minimal effective dose of morphine in current practice may help reduce short‐term side effects and may also improve long‐term outcomes for neonates exposed to opioids in the NICU.^10^

Morphine is most commonly administered through continuous IV infusion, often on central venous catheter (CVC). In 2015, we implemented insertion and maintenance bundles for CVCs in our unit to prevent CVC‐related infections.^11^ The maintenance bundle included limitation of catheter entry 12 by avoiding intermittent drug injection through the CVC and limiting additional ports to the main tubing, that is, reducing continuous drug infusions. A way to achieve such a limitation of catheter entry is to switch IV treatments to the oral route. Oral morphine treatments are available and could theoretically be used in ventilated premature neonates although no published data are available on the use of oral morphine in this population. The available data were obtained in term born neonates showing neonatal abstinence syndrome after birth^13^, ^14^ or in extubated premature infants with a corrected age above 34 weeks,^15^ both contexts being different from continuous analgesia for ventilated neonates.

Although continuous IV morphine is not routinely used for all intubated neonates, as currently recommended,^16^ it is frequently used in our NICU for neonates with expected long duration of invasive ventilation and doses are adjusted using an algometric scale, as recently recommended.^4^ The ComfortNeo scale^17^ is routinely used in our unit to adjust analgesic and/or sedative treatments. As a standard of care, we implemented a continuous IV to oral morphine switch in 2016 and decided to assess this policy's efficacy and efficiency.

The main aims of this study were (a) to compare ComfortNeo pain scores in intubated/ ventilated neonates treated with oral morphine after switching from continuous IV (efficacy); (b) to compare the doses of morphine used by IV continuous versus oral routes (efficiency); and (c) to assess tolerability by collecting all adverse events potentially attributable to morphine and by describing main morbidities at discharge.

We hypothesized that oral morphine would achieve proper pain control and that the same daily dose would be required when using the oral route as compared to IV continuous morphine in intubated and ventilated newborn infants, without severe safety issues.

## 2 METHODS

### 2.1 Study design, setting, and population

We performed a retrospective study in eligible premature infants hospitalized in the NICU of the Centre Hospitalier Intercommunal de Créteil, France. This unit has nine beds dedicated to intensive care and 10 beds dedicated to intermediate care with around 300 admissions each year. Patients were included from January 1, 2016, to December 31, 2017 because a 2‐year span was considered relevant for data analysis after implementation of the protocol in 2016. Patients who received oral morphine during the study period were identified from the local pharmacy's charts.

### 2.2 Participants

#### 2.2.1 Inclusion criteria

To be included, patients had to be intubated, ventilated, and treated with IV morphine followed by oral morphine for at least 48 hours.

#### 2.2.2 Exclusion criteria

Patients were excluded from our study if morphine was used to treat withdrawal syndrome or to provide palliative care.

### 2.3 Data collection

Data were collected retrospectively from medical charts and nurses’ surveillance charts.

The following baseline data were collected: sex, birth weight, gestational age at birth, small for gestational age based on Kramer's growth curves,^18^ multiple pregnancies, delivery route, cause of intubation, weight, and corrected age on the day morphine was switched to the oral route.

### 2.4 Local protocol for morphine administration

For infants with an expected duration of invasive ventilation shorter than 12 hours, there was no systematic analgesia or sedation. But after a 12‐hour span, if the child showed signs of pain or discomfort, including a ComfortNeo scale higher than 14, morphine was introduced as first‐line treatment.

Dedicated morphine sulfate solutions were used for the IV and oral routes. IV morphine was administered continuously through a port to the peripherally inserted central venous catheter. Oral morphine was administered as a bolus via the gastric tube.

At IV treatment initiation, relatively low doses were recommended, depending on gestational age at birth and postnatal age: Loading doses ranged from 0 to 30 µg/kg and continuous doses from 3 to 20 µg/kg/h (see Table S1). Dose adjustments were based on clinical assessment and ComfortNeo scales scores: If the score was less than 10, the attending physician had to consider a decrease in dose, and if the score was greater than 14, the attending physician had to consider an increase in dose.

Oral morphine administration was decision of the attending physician according to the expected duration of invasive ventilation (longer than 72 hours) and patient contraindication to the oral route (such as no enteral feeding or surgery).

Although the bioavailability of oral morphine is considered to be 30% to 50% of IV bioavailability in older children and adults,^19^ it has never been specifically assessed in premature neonates. Two clinical studies^13^, ^15^ and a pharmacokinetic model^14^ estimated a 50% bioavailability in term neonates. In our NICU, we took care to maintain the same dose as the IV prescription when switching to the oral route. When switching to the oral route, the total daily IV dose was thus divided in four administrations to maintain the same total daily dose. For example, a child receiving 10 μg/kg/h IV morphine (ie, 240 µg/kg/d) received 60μg/kg/6h oral morphine. The form used (off‐label) was Oramorph® (Molteni), in vials of 5 mL = 10 mg (2000 μg/mL). The solution was diluted to ensure more precise dosage. For example, 1 mL = 2000 μg of the solution was diluted in 9 mL of sterile water to obtain a solution of 10 mL = 2000 μg, that is, 1 mL = 200 μg. A dose of 60 μg/ kg/6 h in a child of 1000 g therefore corresponded to 0.3 mL of the dilution/6 h via oral route.

Midazolam and paracetamol treatments were added to morphine by decision of the attending physician in order to avoid escalation of morphine doses or if adverse effects of morphine (such as urine retention or slow digestion) were observed. Although midazolam has no analgesic effect and should not be routinely used in ventilated neonates,^20^ it is accepted as additional treatment in our unit when morphine doses are considered too high with persisting signs of pain or discomfort, that are often difficult to disentangle.

### 2.5 Outcomes

The following parameters were collected 24, 18, 12, and 6 hours before the oral switch of morphine; at the time of the oral switch; and then every 6 hours until 48 hours after the oral switch: morphine dose expressed as µg/kg/h based on the daily dose received, ComfortNeo score, mean arterial blood pressure (mm Hg), ventilation mode (conventional mechanical ventilation or high‐frequency ventilation), mean airway pressure (cm H_2_O), FiO_2_, urine retention, feeding interruption, paracetamol use, and midazolam use. The ComfortNeo scale includes seven behavioral dimensions, of which six are actually rated: alertness, calmness/agitation, respiratory response (if intubated) or crying (if extubated), body movement, facial tension, and (body) muscle tone.^17^ Responses to each domain are on a 1 to 5 Likert scale, resulting in total scores ranging from 6 to 30. No formal training was provided for caregivers, nor interrater reliability was assessed, but a printed French version of the ComfortNeo scale was placed at each patient's bedside.

We also collected adverse events (dose error, administration route) and withdrawal syndrome after morphine discontinuation. Withdrawal was assessed using Finnegan's scoring system^21^ every 6 to 8 hours in the 2 days following discontinuation of morphine. Withdrawal was defined as having at least one Finnegan's score > 7.

At discharge from hospital, we collected intestinal perforation or stage II and III necrotizing enterocolitis according to Bell's staging^22^ occurring after the oral switch; bronchopulmonary dysplasia defined according to Walsh's physiological definition^23^; severe retinopathy of prematurity (ROP), defined as stage 3 or more and/or laser treatment; any of the following severe cerebral abnormalities on cranial ultrasonography or MRI at term corrected age: intraventricular hemorrhage with ventricular dilatation (Grade III IVH); intraparenchymal hemorrhage (IPH), defined as a large unilateral parenchymal hyperdensity or a large unilateral porencephalic cyst; or cystic periventricular leukomalacia (PVL), defined as periventricular white matter echolucencies.

### 2.6 Statistical analyses

Data were reported as descriptive summary statistics (n, median, interquartile range, minimal and maximal values). Repeated measures for ComfortNeo scores, morphine doses, mean airway pressure, and FiO_2_ were compared using Friedman's test. The proportions of infants having at least one ComfortNeo score of 14 or higher were compared between the two periods: IV treatment or oral treatment using Fisher's exact test. No imputation of missing data was performed. Analyses were performed using SPSS (v.24, IBM). A *P* value < .05 was considered statistically significant.

### 2.7 Ethics

This retrospective study was based on a standard protocol in the unit. For this type of study, no formal consent was required according to national regulations at that time. The local ethics committee confirmed that the study was conducted in line with national regulatory requirements and approved the study and its publication. All data were analyzed anonymously.

## 3 RESULTS

### 3.1 Population

**Figure 1:**
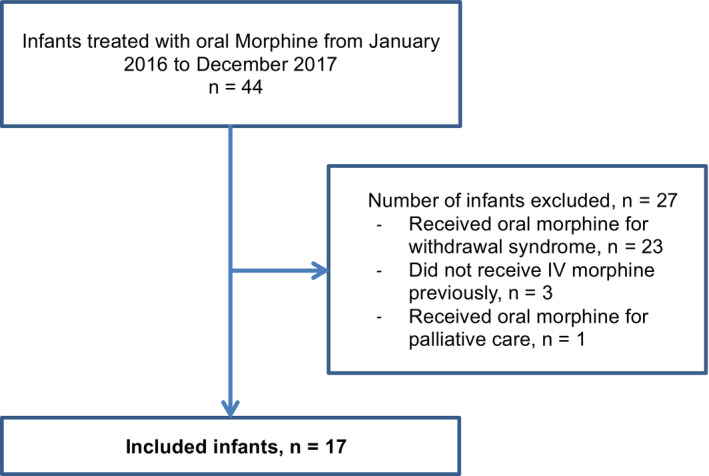
Population flowchart

From January 1, 2016, to December 31, 2017, among intubated and ventilated patients in our unit, forty‐four infants received oral morphine: Twenty‐three infants (52.3%) were treated for withdrawal syndrome, one (2.3%) received palliative care, and three infants (6.8%) were treated for pain symptoms without previous IV treatment. Thus, the remaining seventeen children were included in our study (Figure 1).

Characteristics of the studied population are summarized in Table 1. The median [IQR] gestational age at birth was 25.9 [24.6‐26.9] weeks, and the median birth weight was 670 [620‐750] g. The median postnatal age at oral switch was 30 [22‐36] days.

**Table 1:** Baseline characteristics of the population

Demographic and clinical characteristics of patients, N = 17	
Sex ratio (male/female)	1.4/1
Gestational age at birth (wk)	
Median (IQR)	25.9 (24.6‐26.9)
Range	23.7‐29.6
Birthweight (g)	
Median (IQR)	670 (620‐750)
Range	555‐1315
Small for gestational age, n (%)	6 (35%)
Multiple pregnancies, n (%)	3 (18%)
Cesarean delivery, n (%)	7 (41%)
Cause of intubation, n (%)	
Infant respiratory distress syndrome	7 (41%)
Repeated desaturations and recrudescence of apnea	8 (47%)
Bradycardia	1 (6%)
Surgery	1 (6%)
Postnatal age at the time of oral switch (d)	
Median (IQR)	30 (22‐36)
Range	19‐65
Postconceptional age at the time of oral switch (weeks)	
Median (IQR)	30.4 (27.9‐31.4)
Range	27.1‐38.9
Weight at the time of oral switch (g)	
Median (IQR)	1070 (890‐1230)
Range	745‐2320

### 3.2 Interventions received

#### 3.2.1 Morphine administration

One patient's intravenous treatment had to be resumed 42 hours after the oral switch because of high ComfortNeo scores (=15). All others remained on oral morphine. The median [IQR] duration of oral morphine treatment was 13 [4‐20] days (range 2 to 38 days).

#### 3.2.2 Use of other analgesics/ sedatives

Intravenous midazolam was used in 11 patients at the time of oral morphine switch. Three patients continued to receive IV Midazolam: Two patients were treated more than 48 hours; one patient was treated 24 hours and then switched to oral diazepam. Three patients were switched to oral diazepam simultaneously to the oral morphine switch and were continued on oral diazepam for at least 48 hours. In five patients, midazolam was stopped at the time of the oral morphine switch. Only one patient received oral diazepam after the oral morphine switch, as he/she was not treated with benzodiazepines when receiving IV morphine.

One patient received oral paracetamol (10 mg/kg/6 h) for 24 hours as he/she was treated with IV morphine. Acetaminophen was stopped 6 hours before the oral switch and not resumed after. Two other patients received IV paracetamol (60 mg/kg/d) after the oral switch, including one patient treated for a patent ductus arteriosus.

### 3.3 Efficacy

**Figure 2:**
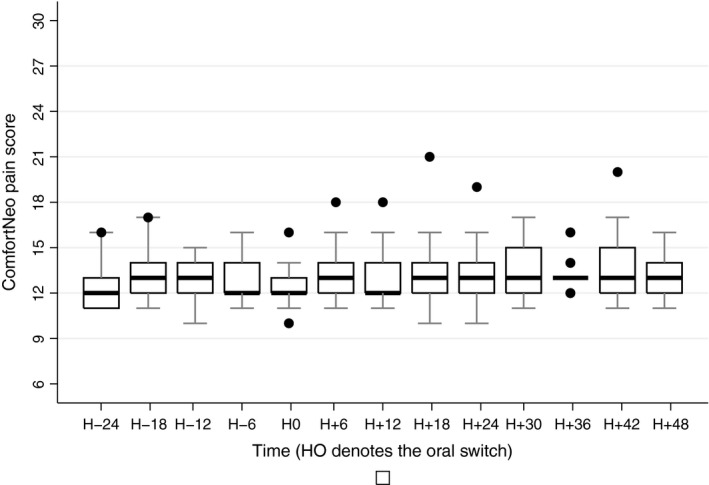
ComfortNeo scores before and after the oral switch. Boxes represent values between the 1st and the 3rd quartile. The bar inside the box denotes median value. The adjacent values are the most extreme values within 1.5 interquartile range of the nearer quartile. Black dots are outliers outside adjacent values

No significant change over time was observed for ComfortNeo scores before and after the oral switch (Figure 2, *P *= .15). The proportion of infants with at least one ComfortNeo score of 14 or higher was 10/17 (59%) during the IV period vs 14/17 (82%) during the oral period (*P *= .26).

### 3.4 Efficiency

**Figure 3:**
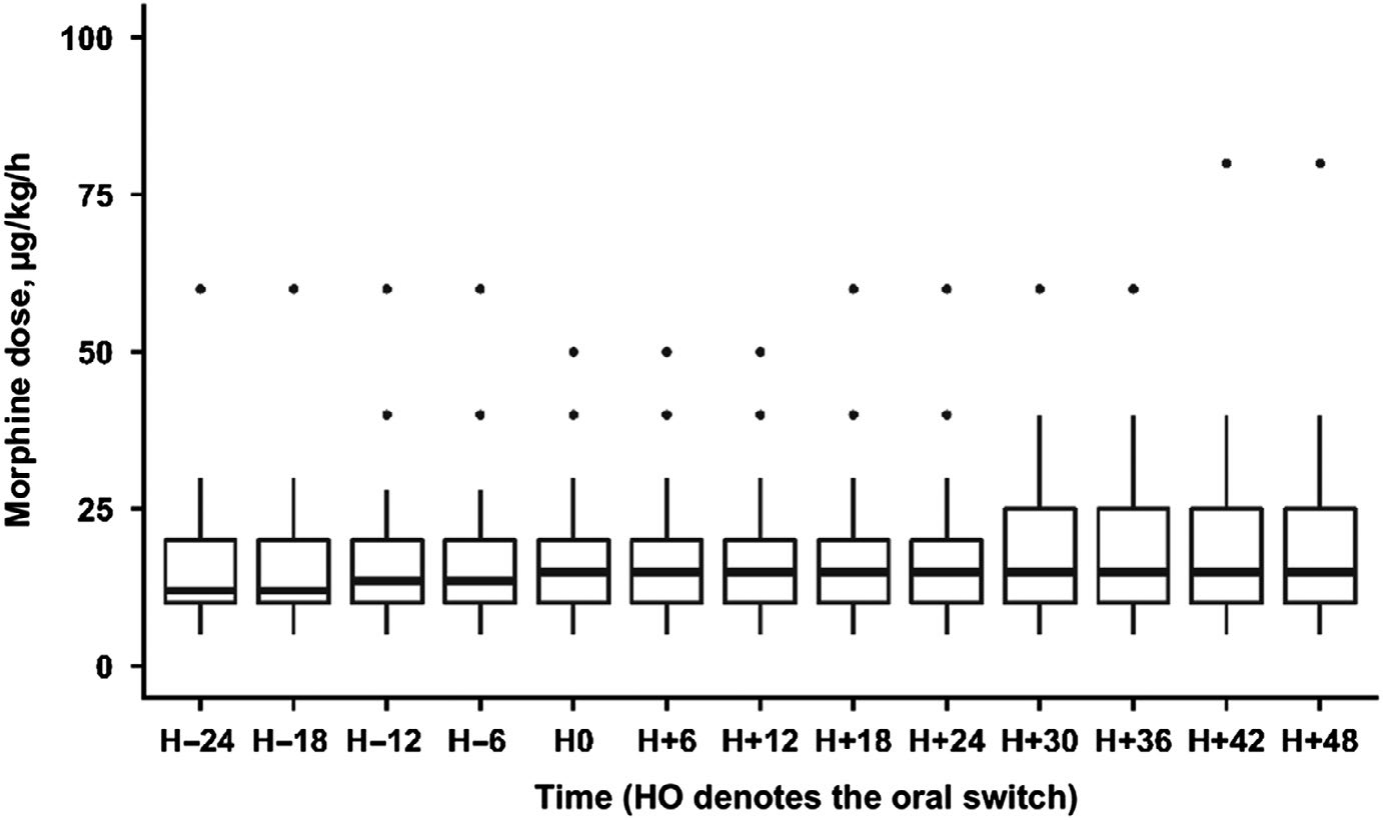
Morphine doses expressed as µg/kg/h before and after the oral switch. Boxes represent values between the 1st and the 3rd quartile. The bar inside the box denotes median value. The adjacent values are the most extreme values within 1.5 interquartile range of the nearer quartile. Black dots are outliers outside adjacent values

Median [IQR] doses were 13.5 [10‐20] µg/kg/h in the IV period and significantly increased to 15 [10‐25] µg/kg/h after the oral switch (Figure 3, *P *= .009). In order to better define the timing of this increase in doses, we performed Friedman's test within each period: IV (before oral switch) and oral route (after the oral switch). During the IV period, no significant change in dose was observed (*P *= .76). During the oral route period, a significant change in doses was observed (*P *< .001).

### 3.5 Tolerability

#### 3.5.1 Ventilation parameters

When comparing periods before and after oral administration of morphine, 15/17 (88%) patients were under identical ventilation mode (conventional ventilation or high‐frequency ventilation). One patient was changed from high‐frequency ventilation before the oral treatment to conventional ventilation after. One patient changed from conventional ventilation before the oral treatment to high‐frequency ventilation after.

#### 3.5.2 Mean airway pressure

No significant change over time was observed regarding mean airway pressures (*P *= .13) with median [IQR] values of 12.4 [10.7‐13] and 10.6 [9.1‐12.5] cm H_2_O for IV and oral morphine courses, respectively.

No significant change over time was observed in regard to FiO_2_ (*P *= .24).

#### 3.5.3 Adverse events

No digestive or urinary adverse event was observed. Only 13 patients had mean arterial blood pressure measurements available. No hypotension, defined as a mean arterial blood pressure below gestational age, was observed. No medication or dosing error was reported.

### 3.6 Withdrawal syndrome

Of the 17 neonates enrolled, withdrawal syndrome occurred in 11 (65%) at oral treatment interruption. The median [IQR] delay of withdrawal syndrome after starting oral morphine was 13 [6.5‐18.5] days (range 3 to 26 days). No withdrawal syndrome occurred in the 48 hours following the oral switch.

### 3.7 Outcomes at discharge from hospital

All infants survived up to discharge. Sixteen (94%) had bronchopulmonary dysplasia; none experienced intestinal perforation or necrotizing enterocolitis; six (35%) had severe ROP; and none had severe cerebral abnormality (15 MRI, 2 head ultrasounds).

## 4 DISCUSSION

The switch from continuous IV to oral morphine did not significantly modify ComfortNeo pain scores in ventilated premature neonates born before 30 weeks of gestation hospitalized in the NICU. However, an increase in daily dose seemed to be necessary in order to achieve an efficacy comparable to that of continuous IV administration. A good tolerability of oral morphine was observed in our study. However, withdrawal syndrome, defined as at least one Finnegan's score > 7, occurred in 65% of patients after oral morphine discontinuation.

Although IV morphine's pharmacodynamics and pharmacokinetics have been extensively studied in neonates,^24^, ^25^, ^26^, ^27^ data on oral morphine in neonates were mainly obtained from infants presenting neonatal abstinence syndrome.^13^, ^28^

In adults, the mean elimination half‐life for morphine ranges from 1.4 to 3.4 hours, so morphine should be administered every 4 hours.^19^ In term neonates up to two months old, morphine's half‐life is 6.5 ± 2.8 hours.^29^ The half‐life in premature infants is 6‐12 hours but is very variable and is inversely related to gestational age at birth.^30^ In our study, we administered oral morphine every 6 hours which is consistent with these pharmacokinetic data. Our results suggest that this time interval was generally appropriate, although inter‐individual variability probably requires specific adjustments for each patient, based on clinical assessment.

Regarding dosage, previous studies in term neonates with neonatal abstinence syndrome have proposed oral morphine doses ranging from 10 to 80 µg/kg/h.^13^, ^28^, ^31^ The POPPI randomized controlled trial comparing oral morphine to placebo for painful procedures in neonates proposed one dose of 100 µg/kg (equivalent of 17 µg/kg/h if administered every 6 hours) based on the British National Formulary for children (2015).^12^ In our study, we used doses ranging from 10 to 25 µg/kg/h which are consistent with above mentioned studies. A neonate specific pharmacokinetic model estimated an oral morphine bioavailability of 48.5% in predominantly term neonates with neonatal abstinence syndrome.^14^ Based on this assumption, a higher dose when switching to oral dose could be proposed. However, in our study, we only observed a 10% increase in median doses with oral administration compared to IV. From this result, we could hypothesize that bioavailability in preterm neonates might be higher than previously reported in term born neonates.^11^ However, as drug elimination can be lengthened in preterm neonates, we may have observed combined effects of previous IV and current oral treatment within the 3 first days of oral administration. A delayed elimination of previous IV morphine is also supported by the progressive increases in oral morphine doses over time in the 3 days following the oral switch. For this reason and because of the uncertainty of bioavailability in premature neonates, it seems safer to administer the same dose at the oral switch (as described in the Method section) and to adjust the dose according to pain assessment during later follow‐up.

We observed a high rate of withdrawal syndrome following oral morphine discontinuation. This is probably related to the relatively long duration of treatment, although we did not collect the duration of IV morphine treatment prior to oral switch. No formal protocol was in place for tapering of morphine doses, which probably increased the frequency of withdrawal syndrome. In consideration of morphine‐induced respiratory depression in neonates undergoing noninvasive ventilation,^32^ morphine was probably sharply discontinued once the patients from our study were extubated. This sudden interruption in treatment probably favored the occurrence of withdrawal. Anticipation of extubation or formal assessment of extubation readiness is required to improve our practice. Safe strategies to prevent withdrawal while maintaining proper respiratory function are needed in the NICU.^33^, ^34^ In addition, in our study, withdrawal syndrome was diagnosed as soon as one Finnegan's score was above 7 whereas the common definition of withdrawal is to have two consecutive Finnegan's scores > 7.^21^ We chose this definition to better reflect our local practice because, in our unit, we do not systematically wait to have two scores to treat the withdrawal syndrome. However, due to this definition, we might have overestimated the occurrence of withdrawal syndrome in our study. In addition, Finnegan's score was developed to evaluate withdrawal in babies of addicted mothers and it might overestimate iatrogenic withdrawal.^35^

Upon hospital discharge, we observed a high rate of bronchopulmonary dysplasia and ROP in our population. These morbidities are probably related to the selected population of infants who received prolonged mechanical ventilation. However, opioids probably prolong mechanical ventilation 3, 16 and the influence of oral morphine on respiratory outcomes should be further assessed. In our study, cerebral imaging at discharge showed no severe cerebral abnormality, although associations have been reported between cumulated morphine doses and poor neurodevelopmental outcome in early childhood.^8^, ^9^ Thus, the safety of this treatment deserves further assessment.

Our study has limitations. First, its retrospective design requires careful interpretation of the results. We could not control for potential biases, including a potential selection bias. Second, while our protocol was part of a strategy aiming to reduce the risk of catheter‐related infections, we did not collect that event. Third, we had a limited number of patients, although this is the largest cohort of premature babies treated with oral morphine to our knowledge. Fourth, our dosing strategy might not be appropriate for younger or less premature infants. Fifth, benzodiazepines were used in 7/17 patients after the oral switch, possibly influencing the results observed on morphine doses and pain scores. Additional research on oral morphine might help improving its use and decrease the use of benzodiazepines, which is currently not recommended in premature infants.^20^ Sixth, nurses were not formally trained to use the ComfortNeo scale and the adherence to our protocol (increase or decrease in morphine doses based on the ComfortNeo score) could not be precisely assessed retrospectively.

Nevertheless, this study offers a simple protocol that might help pain management in this vulnerable population. The efficacy was rigorously assessed using a validated pain scale.^36^ Finally, our clinical observations were consistent with previous pharmacological work in the field.

## 5 CONCLUSION

Continuous IV to oral morphine switch might be useful in ventilated premature neonates born before 30 weeks of gestation and hospitalized in the NICU. Larger prospective studies are required to evaluate required doses, short‐term efficacy, and long‐term safety of oral morphine in this setting.

## CONFLICTS OF INTEREST

All authors have no conflict of interest to disclose.
