# Supplementary material for: Continuous intravenous to oral morphine switch in very premature ventilated infants: A retrospective study on efficacy, efficiency, and tolerability
Source: Paediatr Neonatal Pain. 2020 Jan 3;1(2):45–52. doi: 10.1002/pne2.12011 (PMC8975237; doi:10.1002/pne2.12011)
Supplement: Supplementary file 1 [file PNE2-1-45-s001.docx]

**Supplement: Table S1 Local protocol for morphine doses at IV treatment initiation**

Loading dose as IV bolus over 30 minutes in μg / kg

| Gestational age at birth (weeks) Postnatal age | < 28 | 28-32 | ≥ 32 |
| --- | --- | --- | --- |
| < 3 days | 0 | 5 | 10 |
| 3 – 7 days | 5 | 10 | 20 |
| > 7 days | 10 | 15 | 30 |

Continuous initial IV dose μg / kg / h

| Gestational age at birth (weeks)  Postnatal age | < 28 | 28-32 | ≥ 32 |
| --- | --- | --- | --- |
| < 3 days | 3 | 5 | 8 |
| 3 – 7 days | 5 | 8 | 12 |
| > 7 days | 8 | 12 | 20 |
